# Measurement of wound area for early analysis of the scar predictive factor*

**DOI:** 10.1590/1518-8345.3708.3299

**Published:** 2020-08-31

**Authors:** Adriano Antonio Mehl, Bertoldo Schneider, Fabio Kurt Schneider, Bruno Henrique Kamarowski De Carvalho

**Affiliations:** 1 Universidade Tecnológica Federal do Paraná, Curitiba, PR, Brazil.; 2 Universidade Federal do Paraná, Curitiba, PR, Brazil.; 3 Scholarship holder at the Conselho Nacional de Desenvolvimento Científico e Tecnológico (CNPq), Brazil.

**Keywords:** Wound Healing, Wounds and Injuries, Surgical Wound, Dimensional Measurement Accuracy, Weights and Measures, Software, Cicatrização, Ferimentos e Lesões, Ferida Cirúrgica, Precisão da Medição Dimensional, Pesos e Medidas, Software, Cicatrización de Heridas, Heridas y Lesiones, Herida Quirúrgica, Precisión de la Medición Dimensional, Pesos y Medidas, Programas Informáticos

## Abstract

**Objective:**

to evaluate the use of the 2D-FlexRuler as a facilitating tool for the early calculation of the predictive scar factor of chronic wounds.

**Method:**

a descriptive study with a quantitative, experimental, longitudinal and prospective approach. The sample consisted of 22 outpatients. 32 chronic wounds were analyzed. The wound edges were identified and drawn on the 2D-FlexRuler. The calculations of the areas of chronic wounds were obtained by manual, traditional methods, by software and Matlab algorithm. These areas were compared with each other to determine the efficiency of the proposed ruler in relation to traditional methods.

**Results:**

the calculation of the wound area by the traditional method and *Kundin*’s coefficient show average errors greater than 40%. The manual estimation of the area with the 2D-FlexRuler is more accurate in relation to traditional measurement methods, which were considered quantitatively disqualified. When compared with the reference method, for example, the Klonk software, the data obtained by 2D-FlexRuler resulted in an error of less than 1.0%.

**Conclusion:**

the 2D-FlexRuler is a reliable metric platform for obtaining the anatomical limits of chronic wounds. It facilitated the calculation of the wound area under monitoring and allowed to obtain the scar predictive factor of chronic wounds with precocity in two weeks.

## Abstract (pt)

**Objetivo:**

avaliar o uso da régua 2D-FlexRuler como ferramenta facilitadora para o cálculo precoce do fator preditivo cicatricial de feridas crônicas.

**Método:**

estudo descritivo com abordagem quantitativa, experimental, longitudinal e prospectiva. A amostra foi de 22 pacientes ambulatoriais. Foram analisadas 32 feridas crônicas. As bordas das feridas foram identificadas e desenhadas na 2D-FlexRuler. Os cálculos das áreas das feridas crônicas foram obtidos pelos métodos manuais, tradicionais, por *softwares* e algoritmo Matlab. Essas áreas foram comparadas entre si para determinar a eficiência da régua proposta em relação aos métodos tradicionais.

**Resultados:**

o cálculo da área da ferida pelo método tradicional e coeficiente de Kundin demonstram erros médios maiores do que 40%. A estimativa manual da área com a 2D-FlexRuler é mais exata em relação aos métodos tradicionais de medição, os quais foram considerados quantitativamente desqualificados. Quando comparada com o método de referência, i.e., o *software* Klonk, os dados obtidos pela 2D-FlexRuler resultaram em um erro menor que 1,0%.

**Conclusão:**

a régua 2D-FlexRuler é uma plataforma métrica confiável para a obtenção dos limites anatômicos de feridas crônicas. Facilitou o cálculo da área da ferida sob monitoramento e permitiu obter o fator preditivo cicatricial de feridas crônicas com precocidade em duas semanas.

## Abstract (es)

**Objetivo:**

evaluar el uso de la regla 2D-FlexRuler como herramienta facilitadora para el cálculo temprano del factor predictivo de cicatrices en heridas crónicas.

**Método:**

estudio descriptivo con enfoque cuantitativo, experimental, longitudinal y prospectivo. La muestra consistió en 22 pacientes ambulatorios. Se analizaron 32 heridas crónicas. Los bordes de la herida fueron identificados y dibujados en 2D-FlexRuler. Los cálculos de las áreas de heridas crónicas se obtuvieron por métodos manuales, tradicionales, por *software* y algoritmo Matlab. Estas áreas se compararon entre sí para determinar la eficiencia de la regla propuesta en relación con los métodos tradicionales.

**Resultados:**

el cálculo del área de la herida por el método tradicional y el coeficiente de Kundin muestran errores promedio superiores al 40%. La estimación manual del área con 2D-FlexRuler es más precisa en relación con los métodos de medición tradicionales, que se consideraron descalificados cuantitativamente. Al ser comparados con el método de referencia, es decir, el *software* Klonk, los datos obtenidos por 2D-FlexRuler arrojaron un error de menos del 1,0%.

**Conclusión:**

la regla 2D-FlexRuler es una plataforma métrica confiable para obtener los límites anatómicos de las heridas crónicas. Facilitó el cálculo del área de la herida bajo monitoreo y permitió obtener el factor predictivo de cicatriz de heridas crónicas con precocidad en dos semanas.

## Introduction

Wounds may be classified into acute and chronic^(^ 1 ^)^. Acute wounds are those that have a controlled inflammatory response, responding predictably to the recommended treatment. They heal without complications, usually in three weeks from the beginning^(^ 2 ^)^.

Chronic wounds are defined as those that failed to progress in the ordered and overlapping phases of healing, which are 1) hemostasis and inflammation, 2) proliferation and 3) maturation and remodeling. They remain stationary in the inflammatory phase, despite proper wound management^(^ 2 ^-^ 4 ^)^.

In this case, the healing process does not occur in the expected period and in an orderly manner to restore the anatomical and functional integrity of the injured tissues^(^ 2 ^-^ 3 ^,^ 5 ^)^. After the initial tissue damage, several factors may contribute to the delay in the healing process, either due to the severity of the injury or to the patient’s poor state of health^(^ 6 ^)^. Among the factors related to scar deficit are those considered systemic, such as aging, malnutrition, diabetes, chronic diseases, peripheral vascular disease, sickle cell anemia, vasculitis, use of medications such as corticosteroids^(^ 6 ^)^. Local factors include ischemia, edema and wound infection^(^ 3 ^)^.

There is no pre-established consensus for chronicity, but wounds that do not show a reduction in dimensions after 2 to 4 weeks are likely to become chronic^(^ 2 ^)^.

The main groups of chronic wounds of non-surgical origin are vascular ulcers of the lower limbs, neuropathic ulcers, such as those found in diabetics and pressure injuries^(^ 3 ^,^ 5 ^,^ 7 ^)^.

Surgical wounds can be considered acute when healing happens by first intention and can become complex and chronic when they present complications such as dehiscence and infection, requiring healing by second intention^(^ 4 ^,^ 7 ^)^.

The scar deficit of chronic, surgical and non-surgical wounds is related to the maintenance of prolonged inflammatory activity^(^ 8 ^-^ 9 ^)^ resulting from the abundant infiltration of neutrophils, the presence of high levels of pro-inflammatory cytokines, reactive oxygen species and metalloproteinases^(^ 3 ^)^. This may happen due to a local infectious process, which has an incidence greater than 53%^(^ 10 ^)^ in chronic wounds, as well as by bacterial biofilm^(^ 11 ^)^ present between 60% to 90% of cases of chronic wounds^(^ 8 ^-^ 9 ^,^ 11 ^-^ 12 ^)^.

Biofilms are complex consortia^(^ 8 ^)^ of mixed microbiological ecosystems, formed by populations developed from one or more bacterial species mainly, but also by fungi, viruses and protozoa^(^ 12 ^)^. This microbiota forms an extracellular three-dimensional polymeric substance that may cover the surface of wounds, compromising healing^(^ 11 ^-^ 12 ^)^ and helping to understand the recalcitrant behavior of chronic wounds^(^ 9 ^)^ especially in diabetic, elderly and immobilized patients^(^ 12 ^)^.

About 234 million surgeries are performed worldwide each year. A retrospective study based on data from the US *Wound Registry*, indicated that 20.8% of all types of wounds are represented by surgical wounds that did not heal, with an average cost of treatment for wound healing of US$ 3,927^(^ 7 ^)^. This is an example of how the cost of treating surgical wounds with scar deficits can increase, due to the complexity and longer time of outpatient follow-up^(^ 7 ^)^.

Often disguised as a comorbid condition, the chronic wound represents a vicious cycle and a silent epidemic that affects a large fraction of the world population^(^ 5 ^)^. It imposes a significant and often underestimated burden on the individual, the health system and society as a whole^(^ 3 ^,^ 5 ^)^ due to the significant cost of medical assistance and duration of treatment^(^ 1 ^,^ 13 ^)^.

Chronic wounds have a significant impact on the health and quality of life of patients^(^ 1 ^)^ and their families. They cause pain, loss of function and mobility, depression, anguish and anxiety, embarrassment and social isolation, financial charges, prolonged hospitalizations, chronic morbidity and death^(^ 5 ^)^.

The assessment and documentation of wounds need to be reliable so that health professionals can make a better diagnosis^(^ 14 ^)^, effectively quantify the benefits and results of the therapeutic approaches used^(^ 6 ^)^, identify with greater precocity those who are at risk of non-healing of wounds, which is important for the patient^(^ 14 ^)^.

The methods for evaluating acute and chronic wounds, surgical or non-surgical, include measuring the area, volume and perimeter^(^ 15 ^)^. Each of them has strengths and limitations that lead to their varied use in different clinical contexts.

Estimating the wound area by multiplying the largest linear axis, namely, length C by the largest axis perpendicular edge to edge, that is, the greatest width perpendicular L to the wound area, however, is probably the most popular method among health professionals^(^ 15 ^-^ 16 ^)^. But this estimate using the smallest rectangular area contained in a wound can overestimate the real value in 40%^(^ 16 ^)^. In order to minimize the error of this overestimated calculation, the wound area can be determined using an ellipsoidal geometry^(^ 16 ^-^ 17 ^)^. This approach assumes the largest closed ellipse in the wound and this calculation can be obtained by multiplying the area of the rectangle (C x L) by 0.785 (π / 4).

However, a more accurate way to assess wound healing can be obtained by monitoring the percentage reduction of the area over a given period^(^ 16 ^)^.

The importance of continuous wound assessment, particularly in the first 2 to 4 weeks of treatment, has been highlighted by several studies^(^ 6 ^,^ 14 ^,^ 16 ^,^ 18 ^)^ that demonstrate a correlation between the percentage of reduction in the wound surface area and progress of treatment^(^ 14 ^-^ 16 ^)^. A percentage reduction in the wound area of 10% to 15% *per* week of treatment predicts healing^(^ 19 ^)^. 25% reduction in the wound area within two weeks of treatment^(^ 20 ^)^ or a reduction in the wound area of 20% to 40% within two to four weeks of treatment has proven to be an adequate predictor of healing and a reflection of treatment effectiveness^(^ 16 ^)^. It is recommended that clinical procedures be reassessed if the wound does not reduce the surface area more than 40% within four weeks^(^ 14 ^)^.

The percentage rate of reduction in the area of a wound can be used to distinguish between a wound with potential for healing or not^(^ 16 ^)^ and also as an important tool to distinguish between effective and ineffective treatment regimens^(^ 6 ^,^ 14 ^,^ 16 ^,^ 18 ^)^.

There are several technologies for measuring wounds, both hardware and software^(^ 14 ^,^ 18 ^)^. From a clinical point of view, these technologies have high costs, hindering their easy access^(^ 15 ^)^.

These technologies and approaches must take into account that many wounds have irregular borders, requiring the correct identification of the anatomical limits of the wound, recognizing the tissue flexibility of the cavity or deep wound, as well as the natural curvatures of the human body^(^ 16 ^)^.

A standardized method for measuring wounds, in the absence or minimization of user variability and subjectivity, would allow accurate and reliable documentation that can be compared in different clinical contexts^(^ 14 ^,^ 16 ^,^ 18 ^)^.

This article introduces a new proposal of a transparent, two-dimensional (2D), flexible, low-cost, sterilizable by ethylene oxide, polymeric ruler to be used for wound measurements and data recording in a surgical or non-surgical environment.

However, there is a great variability in the literature on how many weeks the wound areas should be calculated^(^ 6 ^,^ 14 ^-^ 16 ^,^ 18 ^-^ 20 ^)^ in search of the scar predictive factor. Some authors mention that in two weeks of monitoring the area of chronic wounds it is already possible to obtain a predictive factor for healing^(^ 16 ^,^ 19 ^-^ 20 ^)^.

The aim of this study was to evaluate the use of the 2D-FlexRuler as a facilitating tool for the early calculation of the scar predictive factor for chronic wounds.

## Method

This is a descriptive study with a quantitative, experimental, longitudinal and prospective approach.

Patients with wounds who sought care at the outpatient for wounds and diabetic foot, spontaneously or by referral, were evaluated. The recruitment phase aimed to achieve a minimum of 30 chronic wounds for the research. Wounds with more than 4 weeks of evolution were considered chronic^(^ 2 ^)^, regardless of non-surgical or surgical origin.

According to the demand and assessment of each case, patients with chronic wounds who were selected by the researcher were invited to continue the research protocol. They received guidance, read and signed the Free and Informed Consent Form (FICF) and authorization to use the image.

The following criteria were observed for the inclusion of individuals in the research: men and women; adults over 18 years old; patients with chronic non-surgical wounds such as chronic vascular ulcers of the lower limbs, pressure injuries and neuropathic ulcers and chronic surgical wounds with postoperative complications such as dehiscence; non-diabetics and type I or type II diabetics undergoing treatment and medical follow-up; lucid and oriented, verbalizing, walking or using a wheelchair, transport stretcher or need for external supports (cane, crutch or walker) and patients with spinal cord injuries.

For the exclusion criteria of individuals in the research, the following were observed: sedated, unconscious, comatose or obnubilated patients; carriers of debilitating chronic diseases; psychiatric; with difficulty in verbalization; in mechanical ventilation or in need of any kind of external assistance, even if temporary; with external pacemaker; congestive heart failure; chronic kidney disease with dialysis hypotension severe coronary insufficiency; history of seizure; wounds with exposure of metal plate, metal rod, metal screw or metal wire; external fixator on the limb with the wound; undergoing chemotherapy or radiotherapy; corticosteroid pulse; in chronic use of non-hormonal anti-inflammatory drugs; thyroid disease without proper monitoring; using special dressings that for some reason cannot be removed; alcoholic, smokers and drug users; poor hygiene conditions.

In total, 32 chronic wounds were identified in a group of 22 diabetic and non-diabetic patients who were eligible for this research, as they met the inclusion criteria. Therefore, there were patients with more than one chronic wound.

Data collection took place from May 2016 to March 2017 and was performed in a clinic for wounds and diabetic foot in Curitiba-Paraná, Brazil, duly adequate to receive patients with wounds.

Among the criteria that were used to suspend or terminate the research were the patient’s non-adaptation to the evaluation routines, the need for amputation of the limb affected by the wound and death. None of these situations occurred.

Of the 32 chronic wounds, eight were followed for three consecutive weeks and 24 were followed for two consecutive weeks.

Patient assessments were scheduled regularly from the beginning to the end of the proposed follow-up period and performed according to the standard procedure provided in the study protocols.

In the measurements of all wounds, a centimeter, flexible, two-dimensional, transparent ruler was used, called 2D-FlexRuler, which was developed to record the anatomical limits of wounds. To obtain metric data by traditional methods, rigid metric tools such as the triangular metallic scalimeter and a Vernier caliper type 125MEB-6/150 (Starrett, Itu, São Paulo, Brazil) were used.

All materials for dressings and 2D-FlexRuler, already sterile, were offered free of charge by the researcher. He performed all outpatient clinical assessments, dressing changes and wound bed preparations being monitored, and weekly documentation of measurements also for free. For photographic monitoring, a photographic camera was used, positioned perpendicular to the wounds at a focal distance of approximately 50 centimeters between the equipment lens and the wound bed. All the methods used in the measurements were documented photographically. Throughout the collection period, the photographs were always obtained by the researcher who used a GE Brand digital camera, serial number X010061977, Model X5, 14.1 megapixels, with 15X optical zoom and 5.7X digital zoom (combined zoom 85.5X), with flash mode disabled. This monitoring allowed the analysis, at any time, of the values obtained referring mainly to the area and perimeter of the assessed wound.

**Figure 1:**
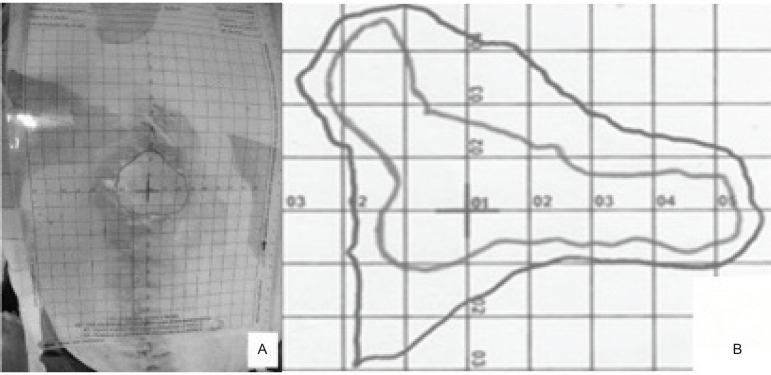
2D-FlexRuler. Curitiba, PR, Brazil, 2017

The 2D-FlexRuler (Figure 1) has the innovation and differential to be formed by two overlapping transparent non-adherent sheets, sterilizable by ethylene oxide, supplied in a sterile envelope, which must be broken only at the time of use. The checkered sheet is 22.0 cm x 17.0 cm with divisions of one centimeter in vertical and horizontal axes, forming 1.0 cm^2^ squares over a total measurement area of 374.0 cm^2^ *per* ruler (Figure 1).

The second, fully transparent, non-centimeter sheet is the one that enters in contact with the wound and is discarded according to the appropriate and current rules.

Because it was developed specifically in A4 size, this ruler already has perforations that allow it to be stored in physical records. It can also be scanned, photographed and digitized. The 2D-FlexRuler allows you to correctly visualize the possible anatomical limits of the wound (bed, edge, margin), even if the wound is in a highly curved region. With a permanent marker or a simple pen, the boundaries of the wound can be drawn on the outer sheet. In addition, the 2D-FlexRuler has an accessory, removable ruler with a 300 mm x 22 mm atraumatic edge incorporated in its right face. This accessory ruler can be used without risk of tissue damage to determine the depth of the bed and, consequently, the wound volume and to assist in the metric reading using a scale also in millimeters.

For each week of monitoring patients with chronic wounds, a new sterile 2D-FlexRuler was used to mark the anatomical boundary of the wound edge.

In Figure 1A, the 2D-FlexRuler is positioned and in contact with the lesion due to abdominal dehiscence. Figure 1B shows the comparison between the first (external line) and the fourth record (internal line) when the centimeter sheets were overlapped.

When obtaining dimensions C and L, the wound area can be estimated using a formula based on the rectangle and corrected by the *Kundin* ellipsoidal coefficient (C x L x 0.785). A manual estimate of the area of the figure can be made by counting the total of 1.0 cm^2^ squares and fragments. A more difficult task is to estimate the perimeter of the wound.

The percentage of wound area contraction over the weekly period (COi) can be calculated by considering the area of the wound surface at the initial monitoring time (S0) and the area of the wound surface at the monitoring week (Si) by equation 1.

Equation 1 $$\mathrm{COi}=100\left(S0-\mathrm{Si}\right)/S0$$

In order to improve the automatic spatial estimation of wounds, in relation to the area and perimeter, the images marked on the ruler can be digitized, stored and transferred to be used sequentially with auxiliary methods, such as software and image processing programs as: Digimizer^(^ 21 ^)^, Klonk^(^ 22 ^)^ and Matlab^(^ 23 ^)^.

Digimizer image analysis software (MedCalc Software bvba, Ostend, Bélgica) is a software package for image analysis that allows precise manual measurements of an object. It supports radiographs, micrographs and all kinds of images in files of jpg, gif, tiff, bmp, png, wmf, fem and *Digital Imaging and Communications in Medicine* (DICOM) format. Images can also be rotated, inverted and filtered. Through this software it was possible to obtain the area and perimeter using a scanned image from 2D-FlexRuler. A calibration must be performed informing the software what is 1.0 cm on the horizontal axis or on the vertical axis, since the ruler has a centimeter grid and each square is equal to 1.0 cm^2^ perfect on the proposed ruler.

The software Klonk *Image Measurement*, produced in Cheyenne, WY, USA, is a planimetric tool for measuring angles, lengths and areas on surfaces. Originally designed for medical research, it can also be applicable for engineering and design. It is possible to work with a wide variety of scanned or imported image formats, such as DICOM files, vector objects, video file frames and RAW camera format. In this study, the determination of the area and perimeter of the wound was performed in exactly the same way as described in the Digimizer software.

Klonk was chosen as the reference standard for this study, after comparing his results in an experiment where three different figures were measured millimetrically and manually.

Matlab, produced in Mathworks, Natick, Massachusetts, USA, is a computing software with a programming language focused on numerical, graphic and technical-scientific computing.

**Figure 2:**
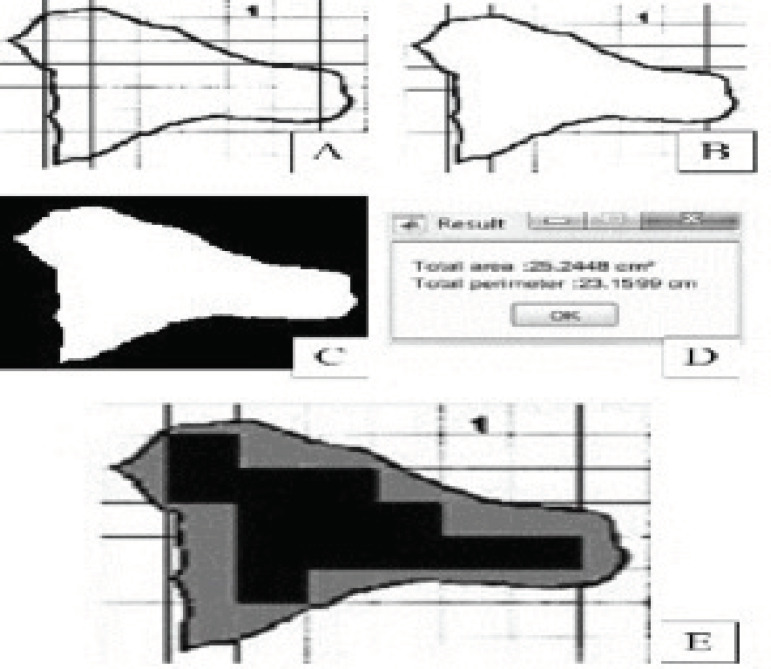
Matlab images to obtain the value of the demarcated wound area. Curitiba, PR, Brazil, 2017

To determine the wound area, an algorithm was implemented using the following basic sequence shown in Figure 2.

The original image of the wound recorded by 2D-FlexRuler was scanned and converted into a black and white image, containing the centimetric line demarcated both outside and inside the wound edge record (Figure 2A).

The rectilinear markers of the centimeter line were excluded from inside the detected edges of the wound (Figure 2B).

After this moment, the exterior is “painted” with black pixels (Figure 2C). The internal border of the wound remains to create a closed white region. The wound area is estimated by integrating the number and geometry (horizontal x vertical dimensions) of the white pixels in this closed white area. The perimeter is calculated using the concept of adjacency between white and black pixels. Again, considering the pixel geometry in the border area where the white pixels belong to the image and the black pixels belong to the background.

Figure 2D shows 25.2448 cm^2^ with the value of the wound area demarcated and 23.1599 cm the value of the perimeter.

This study was approved by the Research Ethics Committee involving Human Beings at the Universidade Tecnológica Federal do Paraná (UTFPR) under nº. 1,606,668 and Certificate of Presentation for Ethical Appreciation (CAAE) nº 56627116.4.0000.5547. The collected data were treated according to the rules of medical confidentiality edited by the Federal Council of Medicine and the UTFPR Research Ethics Committee.

The study followed the Equator Network guide, entitled Revised Standards for Quality Improvement Reporting Excellence (SQUIRE 2.0).

## Results

The selected patients were 11 men and 11 women, with an average age of 62 years old, in the age range of 34 to 77 years old. Nine patients (40.9%) were type 2 diabetics. The accompanied wounds (n = 32) that were in a greater proportion were chronic vascular ulcers of the lower limbs (53.8%). Neuropathic wounds of the diabetic foot were also found with dehiscence and post-amputation scar deficit (25.6%) and pressure injury (12.8%). Of the wounds evaluated, 82% had less than 50.0 cm^2^, 2.5% between 51.0 and 150.0 cm^2^, 5.12% between 151.0 and 250.0 cm^2^ and 10.25% had a surface greater than 251.0 cm^2^.

All patients had their wounds cleaned and prepared with a PHMB solution (polyhexamethylene biguanide). When there was need for debridement to remove devitalized tissues, these were performed before obtaining metrics and photographs. Then, topical agents and topical therapies that contained active principles to control or eliminate biofilm such as PHMB gel, silver and cadexomer iodine were used. There were no complaints by individuals in the study of disabling pain in chronic wounds during the application of the ruler for decaling anatomical limits in the use of topical agents or recommended topical therapies.

The eight wounds that had their anatomical limits recorded for three consecutive weeks, obtaining four readings, were called subset A. The 24 wounds that were followed for two consecutive weeks, obtaining three readings, were called subset B.

The digitalized images of 2D-FlexRuler were manipulated in all three methods aided by computer seeking the estimation of spatial parameters. When three computational methods were compared, no significant difference was noted. The results of this comparison used Pearson’s correlation coefficient among the three methods. The correlation factor between computer aided methods presented Klonk as a reference with the value 1, Digimizer and Matlab with the values of 0.999 and 0.998, respectively.

When 2D-FlexRuler was used, it generated the possibility of obtaining an approximate area by manual estimation, counting the complete square plus the partial square of 1.0 cm^2^. In Figure 2E, thirteen squares filled with 1.0 cm^2^ black are completely surrounded by the demarcated wound area. In addition, it was possible with a small effort to count the partial area totaling about 11.8 cm^2^ of the other squares formed by the gray area. This has produced an area of 24.8 cm^2^.

For the same wound in Figure 2, Table 1 illustrates the difference among manual estimation, computer aided techniques, traditional practices and application of the *Kundin* ellipsoidal coefficient. The differences are relative to the Klonk value, here considered the standard.

**Table 1:** Comparative results with Klonk as standard. Curitiba, PR, Brazil, 2017

Employed technique	Area (cm^2^)	Difference (%)
Klonk*	24.57	Standard*
Manual estimation	24.80	+0.94
Digimizer	24.34	-0.94
Matlab	25.24	+2.23
Caliper and *Kundin*	32.67 (6.5 x 6.4 x 0.785)	+32.97
Traditional ruler	37.5 (5.0 X 7.5)	+52.63

* Klonk standard value

Table 2 shows the errors of the methods when compared with the chosen reference method (Klonk).

**Table 2:** Percentage errors among traditional methods for measuring wounds and *software* analysis. Curitiba, PR, Brazil, 2017

Wounds	Digimizer	Matlab	Caliper *Kundin*	Rigid Ruler *Kundin*
A	1.44	-2.60	-1.78	-16.07
B	2.63	5.41	-24.01	-25.66
C	1.88	0.99	-62.72	-38.86
D	3.76	-0.80	-195.16	-195.16
E	0.99	-2.75	-32.15	-32.98
F	0.94	-0.73	-35.12	0.45
G	3.00	-0.31	-16.71	-30.90
C	-6.60	-6.06	8.54	-18.44
Mean Error %	1.01%	-0.86%	-44.89%	-44.70%

The wounds of subset B were used with subset A to compare the area estimation of the methods with the Klonk method. The results of these relative average errors are 2.86% for Matlab, 96.78% for rigid ruler, 141.8% for Vernier’s caliper and 188.58% for the scalimeter.

**Figure 3:**
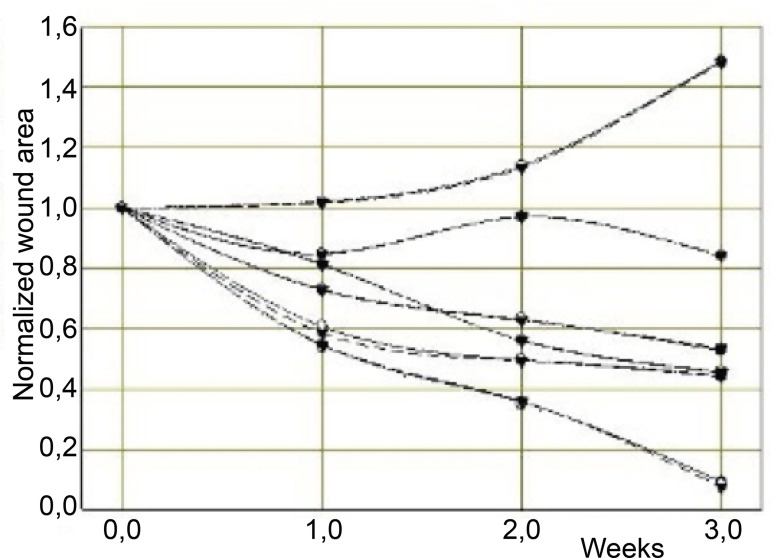
Normalized monitoring of the wound areas of subset A in three weeks. Curitiba, PR, Brazil, 2017

Figure 3 exemplifies six wound areas in subset A that were normalized, using computer aided area estimation by the three computational methods^(^ 21 ^-^ 23 ^)^.

The curves in Figure 3 show the tendency to reduce wound areas after the second reading (between the first and second weeks of monitoring).

The upper curve was an exception and shows the behavior of a wound that, even recovering the lost tissue volume, presents an increasing area due to deep wound tissue flexibility after debridement^(^ 16 ^)^. The same process occurred in the second curve, but there was a recovery in the tendency to reduce the area between the second and third weeks.

## Discussion

For manual estimation, 2D-FlexRuler is much more relevant than traditional measurement methods. It is flexible, sterilizable and allows the registration of wounds on curved surfaces. The manual estimate summarized in Table 1, when compared with the reference method, i.e., the Klonk software resulted in an error of less than 1.0%.

Although the traditional ruler method (caliper, scalimeter and *Kundin*’s ellipsoidal coefficient) has predictive power for qualitative evaluation of the healing potential, mean errors greater than 40% are found, as shown in Table 2, which weakens the use of this method in quantitative analyzes. This magnitude of error had already been reported in the literature^(^ 16 ^)^.

The Klonk and Digimizer software and the Matlab algorithm showed a Pearson’s correlation factor of at least 0.998 with each other. In the Klonk and Digimizer software however, the image limits must be edited by the user, requiring additional effort and a certain skill to obtain the appropriate estimate. On the other hand, the additional image processing algorithms implemented in Matlab showed 0.998 Pearson’s correlation factor, performing edge detection autonomously and, therefore, adding no user effort to demarcate the wound boundary.

When using the larger set of 32 wounds, with patients followed for two weeks and three metric values obtained, the relative mean error of the estimated areas was 2.86% for Matlab analyzes, 96.78% for rigid ruler, 141.8 % for Vernier’s caliper and 188.58% for the scalimeter, leading to the conclusion that they are qualitatively useful, but quantitatively disqualified.

The limitations of the study in relation to the use of 2D-FlexRuler were found mainly in obtaining the tracing of highly exudative and circumferential wounds of the lower limbs. Specific precautions such as reducing the amount of exudate in the wounds to avoid slipping between the wound bed and 2D-FlexRuler sheet “B” when obtaining the wound limits were necessary. The use of a caliper and a scalimeter to measure wounds also brought some technical difficulties, as these instruments are rigid, which made it difficult to obtain data on wounds in regions with anatomical curvature.

The need for continuous monitoring of chronic surgical and non-surgical wounds in diabetics and non-diabetics is reiterated. We encourage the use of the percentage rate of reduction of the wound area obtained in the first two consecutive weeks of treatment as a predictive factor for the healing of chronic wounds, in diabetics and non-diabetics, as described in the literature^(^ 16 ^,^ 19 ^-^ 20 ^)^. Thus, adjustments in the therapy of chronic wounds can be performed with greater precocity in order to obtain better results and a lower rate of complications.

The study demonstrated that the 2D-FlexRuler rule contributes with simplicity, safety, low cost and as a reliable and reproducible documentation for manual wound measurement. The unit value was around US$ 1.0 which can be much lower if scale production is considered. It can be used in children and adults, in outpatients, hospitals, home care services and others.

The data obtained with this ruler can be stored directly in the medical records and can be easily digitized.

Statistical tests on the progression of healing may be more reliable with the metric data obtained with the methodology, stimulating the research that requires evolutionary and comparative measurement of wounds of the most varied causes and complexities.

The application of 2D-FlexRuler in Forensic Medicine is foreseen through the analysis of injuries in victims of aggressions or in *post mortem* injuries.

The estimation of the computer-assisted area was implemented with less complexity when the 2D-FlexRuler was used, since it is already centimetric, already serving as a dimensional template for image softwares.

The use of 2D-FlexRuler in low-level laser therapy is also possible for the indication and delimitation of the quadrants that indicated the points of application of the laser in the treatment of wounds.

The developed Matlab algorithm is simple and can be implemented in portable devices, such as cameras and smartphones, to contribute enormously to an initial and reliable assessment of the wound and patient follow-up.

## Conclusion

The 2D-FlexRuler is a reliable metric platform for obtaining the image of the anatomical limits of chronic wounds. It facilitates the calculation of the area of the wound under monitoring, which allowed to obtain the scar predictive factor of chronic wounds with precocity, in two weeks, with more accurate results than those obtained with traditional methods. It can be digitized and used as a new tool in clinical practice and wound research.
